# VNF Chain Placement for Large Scale IoT of Intelligent Transportation

**DOI:** 10.3390/s20143819

**Published:** 2020-07-08

**Authors:** Xing Wu, Jing Duan, Mingyu Zhong, Peng Li, Jianjia Wang

**Affiliations:** 1 School of Computer Engineering and Science, Shanghai University, Shanghai 200444, China; xingwu@shu.edu.cn (X.W.); duanjing@shu.edu.cn (J.D.); ms_nymph@shu.edu.cn (M.Z.); 2 Shanghai Institute for Advanced Communication and Data Science, Shanghai University, Shanghai 200444, China; 3 School of Computer Science and Engineering, University of Aizu, Fukushima 965-8580, Japan; pengli@u-aizu.ac.jp

**Keywords:** virtual network function, placement, border node, subgraph, intelligent transportation

## Abstract

With the advent of the Internet of things (IoT), intelligent transportation has evolved over time to improve traffic safety and efficiency as well as to reduce congestion and environmental pollution. However, there are some challenging issues to be addressed so that it can be implemented to its full potential. The major challenge in intelligent transportation is that vehicles and pedestrians, as the main types of edge nodes in IoT infrastructure, are on the constant move. Hence, the topology of the large scale network is changing rapidly over time and the service chain may need reestablishment frequently. Existing Virtual Network Function (VNF) chain placement methods are mostly good at static network topology and any evolvement of the network requires global computation, which leads to the inefficiency in computing and the waste of resources. Mapping the network topology to a graph, we propose a novel VNF placement method called BVCP (Border VNF Chain Placement) to address this problem by elaborately dividing the graph into multiple subgraphs and fully exploiting border hypervisors. Experimental results show that BVCP outperforms the state-of-the-art method in VNF chain placement, which is highly efficient in large scale IoT of intelligent transportation.

## 1. Introduction

Network Function Virtualization (NFV), fog computing and Internet of Things (IoT) constitute key enablers [1] of intelligent transportation ecosystems. Only the symbiotic orchestration among these enablers would allow the intelligent transportation to be flexible, agile and efficient in deploying multiple services. NFV facilitates the implementation of network functions as a software application running on a non-dedicated hardware in the form of Virtual Network Functions (VNFs) [2]. However, the VNF placement [3,4] in the Edge–Fog–Cloud interplay [5] enabled large scale IoT of intelligent transportation is a challenging problem because edge devices are dynamic and low latency is required by intelligent transportation applications. To face the challenge, a VNF chain placement is proposed for large scale IoT of intelligent transportation meeting resource and time constraint requirements.

Intelligent transportation, playing an important role in smart cities [6], can be defined as the integration of modern technologies, innovations and management strategies in transportation systems to provide enhanced services associated with different modes of transport and traffic management [7]. The IoT of intelligent transportation integrates data collection, communication and processing across transportation systems, allowing for dynamic real-time interaction. Sensors and actuators embedded in vehicles and pedestrians are linked through networks with evolving topology according to their dynamic movements.

**Figure 1:**
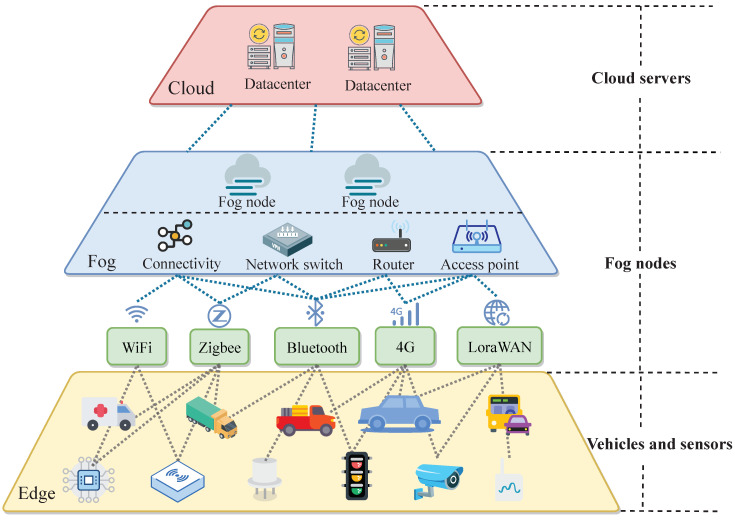
Holistic view of Edge–Fog–Cloud interplay framework for intelligent transportation.

**Figure 2:**
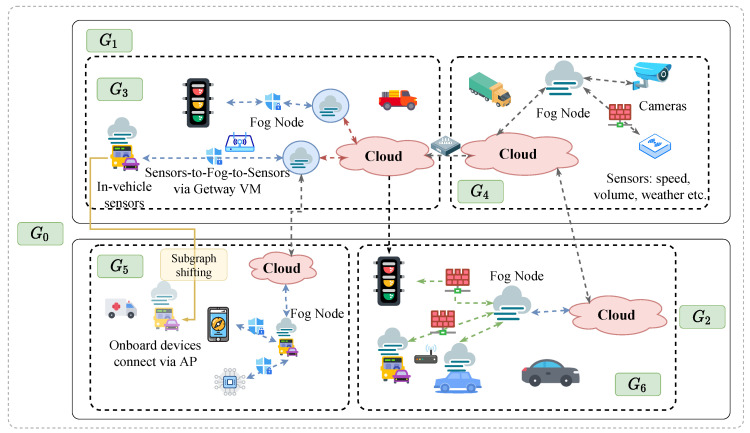
The intelligent transportation networks requiring VNF chain deployment.

Cloud computing infrastructure is indispensable for intelligent transportation systems that sense the environment and transmit a large amount of data to the cloud for processing to achieve service reliability, whereas fog computing extends the cloud computing to the edge of the network to provide low-latency and location-aware services, which moves the execution of real-time applications on devices in proximity of the physical systems with wide-spread geographical distributions. For intelligent transportation, there are various requirements for computing and network resources. On the one hand, some traffic applications need the aggregated and analyzed data in the shape of actionable intelligence to enable some devices to take fast actions. On the other hand, some traffic applications need all data to be stored and analyzed in the cloud but the operating personnel only want that bit of data traveling across networks to ensure security. According to the different amount of data generated by edge devices and the different types of network services that are required to be applied, Service Chains (SCs) must be appropriately deployed in the Edge–Fog–Cloud infrastructures. There have been SDN-assisted frameworks for Edge-Cloud interplay [8,9] and service chain placements with bottleneck removal for Edge–Fog–Cloud interplay [10]. Figure 1 demonstrates a holistic view of proposed Edge–Fog–Cloud interplay framework, which can be implemented as a hierarchical structure with three layers: Edge layer, fog layer and cloud layer. Edge devices, fog nodes and cloud servers should follow NVF management. The proposed Edge–Fog–Cloud interplay framework could be mapped to a graph as demonstrated in Figure 2, which is a flat illustration of the smart transportation networks requiring VNF chain deployment.

The service chain placement in intelligent transportation has its unique requirements because many edge devices in transportation are characterized as distributed in locations and dynamic in mobility, such as vehicles, cyclists, pedestrians, as well as sensors and actuators attached to them. Accordingly, there is a high demand for low-latency and context-aware network services in a flexible and efficient manner. NFV has been emerging as a networking technology to provide flexibility and agility in the deployment of network services, which is agile and flexible in the face of dramatically changing requirements for various services. However, placing VNF chains in Edge–Fog–Cloud interplay is a challenge aiming at reducing time-to-response, end-to-end latency and unnecessary traffic of the backbone network. To meet the challenge, we propose a novel VNF placement algorithm called BVCP (Border VNF Chain Placement) for the Edge–Fog–Cloud interplay in intelligent transportation, which is ideal for dynamic service chain deployments to ensure communications and data processing between vehicles, access points, and traffic lights efficiently. After mapping the dynamic network topology to a graph, the proposed BVCP can divide the graph into multiple subgraphs, so that local network topology changes will not affect the whole topology. In terms of VNF chain placement, BVCP makes full use of border hypervisors in order to complete the VNF placement task efficiently in large scale IoT of intelligent transportation.

1. An Edge–Fog–Cloud interplay paradigm is proposed for the evolving topology of networks in intelligent transportation and it is mapped to a graph.
2. A novel VNF chain placement algorithm called BVCP is designed, which fully utilizes the salient features of subgraphs and border hypervisors to enable flexible deployments of service chains on dynamically evolving IoT networks.
3. The proposed BVCP completes in O(|V|) time, which exactly fits in large scale IoT of intelligent transportation to empower quick intelligent transportation applications and services.

## 2. Related Works

From the perspective of Internet of Things, the intelligent transportation system should be capable of collecting a large amount of data with IoT infrastructure and producing real-time useful information to travelers to guide them to destinations in the most efficient way possible. IoT can be used with the existing intelligent transportation infrastructure for the design of an efficient public transportation system to facilitate the relief of traffic congestion [11]. Sensor technology, an indispensable part of IoT, can be integrated with the transportation infrastructure to collect related data, in which safety, traffic control, and infotainment applications can benefit from multiple sensors deployed for different transportation services [12]. Because of the growing volume of connected sensors and IoT devices in intelligent transportation systems, there is an emergent need for flexible communication and computing resources allocation to remove the bottlenecks in terms of data transmission overhead and data processing latency. Fog computing [5] enables an efficient solution to the problem of transmission overhead and processing latency in IoT, which depends on devices on the edge of the network that have more processing power than the end devices, and are nearer to these devices than the more powerful cloud resources. In the dynamic Internet of Vehicles (IoV) environment, a cloud and fog architecture [13] for real-time intelligent transportation big data analytics was proposed, which merges three dimensions including intelligent computing dimension, real-time big data analytics dimension, and IoV dimension. Another context-aware fog computing framework [14] for intelligent transportation was presented, which consists of multiple intelligent tiers: Internet of Things tier, fog service tier, and global cloud service tier supporting edge analytics for ITS services in a connected car environment. For smart traffic control, the traffic data could be processed right at the edge devices rather than at a centralized facility [15]. However, there are few effective Edge–Fog–Cloud interplay paradigms for the evolving networks in intelligent transportation.

IoT and network softwarization are becoming core enabling technologies of information systems and network management for intelligent transportation. Software-Defined Networking and Network Function Virtualization are expected to support different types of services, including intelligent transportation. Soua et al. discussed the features of multi-access edge computing for vehicular networks and the need of SDN and NFV, to fulfill the requirements in terms of reliability, responsiveness and resiliency [16]. Malandrino et al. built and emulated a vehicular collision detection system, using the Mininet and Docker tools to verify the compatibility of strict delay requirements with SDN and NFV [17]. Copeland et al. proposed an automotive virtual edge communicator with vehicular inter-agent service orchestration and resourcing, which allowed emergency and essential services to pool together resources for vehicles and to provide temporary access nodes and virtualization capacity [18]. Nobre et al. investigated a fog-enabled vehicular SDN focusing on the systems, networking, and services, and they evaluated these design principles for fast traffic accident rescue in emergency vehicles use cases [19]. Based on a distributed Multi-Access Edge Computing architecture, a dynamic placement of the VNFs was provided to manage network traffic, which integrated heterogeneous radio technologies with the vehicular sector to create isolated network slices without risking the core network scalability [20].

Intelligent transportation services are usually composed of various VNFs (e.g., firewall, WAN optimizer, network translation service) based on application demands. The ordered sequence of VNFs forms a service function chain also known as VNF chain. There have been several researches concerning VNF chain placement. In NFV-based Vehicle-to-everything (V2X) networks, a clustered VNF chaining scheme is proposed, which deployed VNFs in clusters according to the cluster head of each vehicle clusters with the expression of the average service time [21]. Chen et al. proved that the problem of NFV chain placement in edge computing environments is NP-complete, and they proposed a metric that can better measure the balance condition of the physical resources [22]. Sun et al. proved NFV service chain deployment is NP-hard with an integer linear programming model to minimize the total service chain deployment cost, and they proposed a time-efficient heuristic based on affiliation-aware VNF placement [23]. Zou et al. proposed a mathematical model to virtualize resource mapping [24]. A deployment algorithm was proposed for NFV chains that optimized performance by minimizing the actual cost of virtual switching [25]. Xu et al. formulated the overall cost of network service chain placement as the combination of setup cost and operation cost and the service chain placement was further formulated as an integer linear programming model with the objective of minimizing the overall cost of setup and operation. Accordingly, they proposed a delay aware dynamic programming based network service chain placement scheme for large networks [26]. However, effective placement of VNF chains is a complex, yet the important challenge to overcome in intelligent transportation in which vehicles and pedestrians, as the main types of edge nodes in IoT infrastructure, are on the constant move.

Enlightened by the network topology extraction and decomposition with graph theory [27,28], an Edge–Fog–Cloud interplay paradigm is proposed for the evolving topology of networks in intelligent transportation and it is mapped to a graph. Furthermore, we present a VNF chain placement algorithm called BVCP to empower flexible deployments of service chains on dynamically evolving IoT networks of intelligent transportation.

## 3. BVCP Method

### 3.1. Problem Formulation

Fog computing has emerged as a promising infrastructure to support intelligent transportation applications. As illustrated in Figure 1, three types of layers, namely edge layer, fog layer and cloud layer are in the considered holistic view of Edge–Fog–Cloud interplay framework. NVF management are to carefully orchestrate function placement on Edge devices, fog nodes and cloud servers.

**Figure 3:**
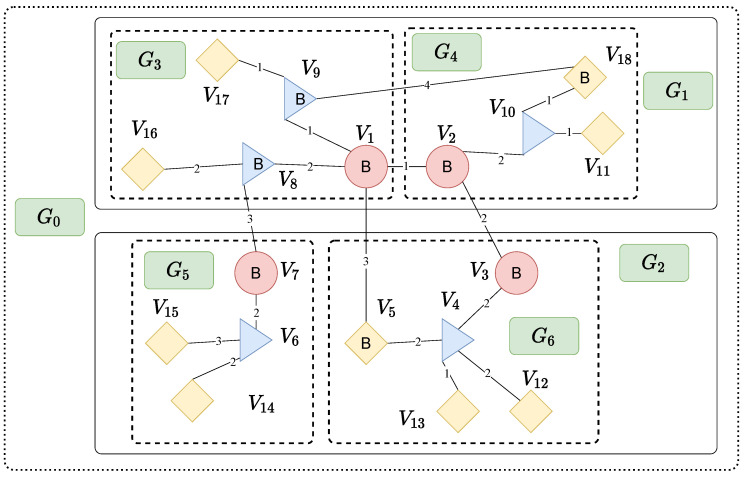
An example of the BVCP graph. Cloud nodes are marked in pink, fog nodes are marked in blue and edge nodes are marked in yellow.

Edge layer is composed of vehicles, cyclists, pedestrians, as well as sensors and actuators attached to them, etc., which are dispersed widely but in the nearest proximity to the physical world. Fog layer is located between the Edge layer and the Cloud layer to play the same or part of the roles of cloud computing. Data collection and sharing from the Edge layer to the Fog layer could be short-range and long-range communication technologies such as low power Wi-Fi, LoWPAN, RFID, NFC, Sigfox, LoraWAN, etc. Each fog node is an autonomous system managing a given set of computational resources. Cloud layer consists of high-geared servers and data centers that are responsible for complicated data analytics and long term data storage. Three layers are integrated together for the proposed Edge–Fog–Cloud interplay to support various intelligent transportation applications. For the pedestrians, vehicles and other entities in the flat illustration of the smart transportation networks requiring VNF chain deployment (Figure 2), they can be abstracted into nodes in the graph as shown in Figure 3. Nodes of different colors represent their position in the Cloud-Fog-Edge hierarchy, yellow for edge devices, blue for fog nodes, and pink stands for cloud servers.

At any point, for a intelligent transportation system to adjust the traffic flow dynamically, online service chain placement is needed. A service chain is composed of a series of VNFs (e.g., firewall, encryption, decryption). Thus, we propose the BVCP to address the VNF chain placement problem.

The major challenge here is that in smart transportation networks, vehicles, as a main type of edge nodes, are on the constant move. Hence, the topology of the network is changing rapidly over time and the service chain may need reestablishment frequently. Notice how the vehicle moves from subgraph $G_{3}$ to subgraph $G_{5}$ in Figure 2 (marked with “subgraph shifting”). Existing methods mostly deal with static graphs, and any changes of the graph typically requires extensive resources.

Formally, we define the following notations. G=〈V,E〉 is a graph which *V* is the hypervisor set and *E* is the edge set. (u,v) is a pair of entry-exit points. $PR\;=\{VNF_{i}\}$ is an online VNF placement requests. the latency constrain and the computational constrain (CPU, RAM, etc.) are defined as *t* and $C=\{C_{i}\}$. The objective is to place the VNF chain on hypervisors in *V* while not violating the constrains *t* and *C*. The objective must be fulfilled with low latency considering the time sensitive nature of intelligent transportation, given the fact that the local topology of the network is changing frequently.

The proposed BVCP addresses this problem by dividing the graph into multiple subgraphs so that local topology changes can be handled easily. In terms of placement, the BVCP method fully exploits the border hypervisors in order to complete the VNF chain placement task efficiently.

### 3.2. Data Structures

In this subsection, BVCP graph, border hypervisor and BVCP tree are defined in detail, which has the following advantages:

- The BVCP graph is compatible with local topology changes.
- The BVCP tree is able handle local topology changes efficiently.
- The space complexity of BVCP is only O(|V|log|V|+|E|) where |V| is the vertex number and |E| is the edge number of the graph.

#### 3.2.1. BVCP Graph

Figure 3 is an example of BVCP graph. All nodes are classified into three categories: Cloud node, fog node and edge node. In Figure 3, cloud nodes are marked in pink, fog nodes are marked in blue and edge nodes are marked in yellow. Edge length denotes network latency between vertices. Vertex, node and hypervisor are used interchangeably during the explanation.

An BVCP graph has local tree-like structures in which the parent node is an upper layer node and the child node is a lower layer node. Within these local area, there are little edges since trees are very sparse graphs. Hence, it is efficient to run Dijkstra algorithm. Such pattern is violated by edges such as $\left(V_{2},V_{3}\right)$. However, logically speaking, tree-like structures would be the main type of structures following the definition of Edge–Fog–Cloud computing paradigm as demonstrated in Figure 1.

The intelligent transportation network graph *G* can be divided into a set of subgraphs, i.e., $G_{1}=\langle V_{1},E_{1}\rangle ,G_{2}=\langle V_{2},E_{2}\rangle ,\ldots ,G_{n}=\langle V_{n},E_{n}\rangle$ that satisfied:

- $\cup_{1\leq i\leq n}V_{i}=V$;
- $\forall i\neq j,V_{i}\cap V_{j}=\emptyset$;
- $\forall u,v\in V_{i},\;\mathrm{if}\;\left(u,v\right)\in E,\left(u,v\right)\in E_{i}$.

The partition is designed to be compatible with fast-moving edge hypervisors like vehicles and pedestrians that move from one subgraph to another. It is not required that the subgraph is strictly a tree as long as each subgraph is sparse. Hence, when a new hypervisor is added into the graph, the partition does not have to change. The network partition has proved to be a NP-Hard problem. However, there are a lot of heuristic algorithms aiming to give satisfying result for this problem in polynomial time, e.g., multilevel graph partitioning [29]. In Figure 3, *G* is split into four subgraphs $G_{3},G_{4},G_{5},G_{6}$. A supergraph is the union of other subgraphs. In Figure 3, $G_{1}$ is the supergraph of $G_{3},G_{4}$ and $G_{2}$ is the supergraph of $G_{5},G_{6}$.

#### 3.2.2. Border Hypervisor

A border hypervisor is an endpoint of a bridge edge that connects two subgraphs. Given a subgraph $G_{i}=\langle V_{i},E_{i}\rangle$, a pair of hypervisors u,v, if $\left(u,v\right)\in E,\;u\in V_{i},\;v\notin V_{i}$, *u* is a border hypervisor of $V_{i}$. In Figure 3, border hypervisors are marked with capital letter B. Fast-moving edge nodes are not considered as border hypervisors.

Border hypervisors have salient features for the VNF chain placement. On the one hand, for those border hypervisors that are not cloud nodes, they represent shortcuts between subgraphs. Intuitively, it would be efficient to put VNFs on them because such placement avoids going all the way up to the cloud layer. On the other hand, to leave one subgraph and enter another subgraph, the path must include one border hypervisor. Hence, it is possible to use dynamic programming to find the shortest path efficiently. We will discuss how to utilize border hypervisors in detail in Section 3.3.

In this paper, B(G) indicates the border hypervisor set of graph *G*.

#### 3.2.3. BVCP Tree

**Figure 4:**
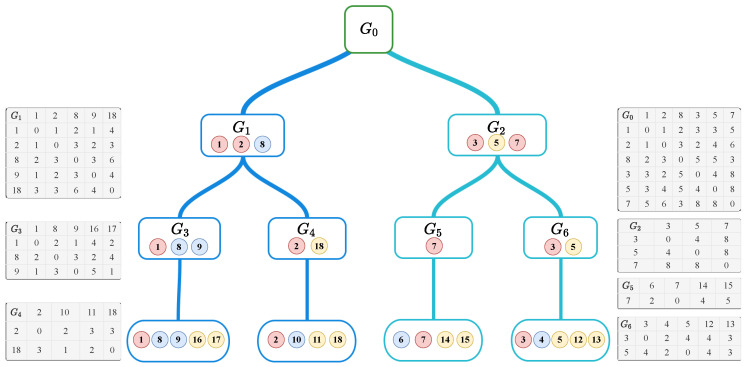
An example of BVCP tree.

To avoid ambiguity, nodes in BVCP tree are always referred as tree nodes, leaf nodes or non-leaf nodes, hypervisors are referred as vertex or nodes. Figure 4 illustrates the corresponding BVCP tree of the BVCP graph in Figure 3. A BVCP tree has the following characteristics:

- The tree is balanced.
- Each tree node is a subgraph. The parent of one subgraph is its supergraph.
- Each non-leaf node has most *a* child tree nodes and each leaf node has most *b* hypervisor nodes.
- Non-leaf nodes maintain a square shortest distance matrix in which the rows and the columns are the border hypervisors while leaf node maintain a non-square matrix where the row is border hypervisors and the column is every hypervisor in that subgraph.
- All leaf tree nodes are at the same level.

In Figure 4, a=2 and b=5, the graph $G_{0}$ is partitioned into two subgraphs $G_{1}$ and $G_{2}$. Furthermore, $G_{1}$ is partitioned into $G_{3}$ and $G_{4}$ while $G_{2}$ is partitioned into $G_{5}$ and $G_{6}$.

From the definitions above, it is easy to conclude that “sub- graph shifting” phenomena only affect leaf nodes. Therefore, when a vehicle moves from one subgraph to another, only the topology of leaf nodes will change. Hence, it is not necessary to update the entire topology, instead, only the leaf node has to be updated. As analyzed in Section 3.2.1, it is easy to deal with subgraphs with Dijkstra’s algorithm, and the time complexity is $O(b^{2}logb)$ which is considered as a small constant. As a result, The BVCP tree is able to handle local topology changes efficiently.

#### 3.2.4. Space and Time Complexity

The space overhead needed for BVCP graph and tree is discussed in this subsection. The overall space overhead needed is SP(EFCgraph)+SP(EFCtree) where SP denotes space needed.

For BVCP graph, the space needed is SP(EFCgraph)=O(|V|+|E|).

The number of tree nodes is $O(\frac{\left|V\right|}{b})$, and the height of the BVCP tree would be $\log_{a}\frac{\left|V\right|}{b}\;+\;1$.

The *a*-partition of a graph containing *N* vertices contains totally $O(log_{2}a\cdot \sqrt{N})$ borders. Each of *a* children share these borders. The average vertex number at level *i* is $\frac{\left|V\right|}{a^{i}}$. Consider a tree node at level i−1: It has $\frac{\left|V\right|}{a^{i-1}}$ vertices and $O(log_{2}a\cdot \sqrt{\frac{\left|V\right|}{a^{i-1}}})$ borders; for each child at layer *i*, the border number would be $O\left(log_{2}a\cdot \sqrt{\frac{\left|V\right|}{a^{i}}/a}\right)=O\left(log_{2}a\cdot \sqrt{\frac{\left|V\right|}{a^{i+1}}}\right)$ since they share all border vertices. Hence, at a non-leaf node, the space needed for the distance matrix is its square, that is $O(log_{2}^{2}a\cdot \frac{\left|V\right|}{a^{i+1}})$. There are $a^{i}$ tree nodes each layer and $\log_{a}\frac{\left|V\right|}{b}\;+\;1$ layers, so the space overhead needed for non-leaf nodes is $O(log_{2}^{2}a\cdot \frac{\left|V\right|}{a}\cdot \log_{a}\frac{\left|V\right|}{b})$.

In particular, for leaf node in a BVCP tree, $O\left(log_{2}a\cdot \sqrt{\frac{\left|V\right|}{a^{i+1}}}\right)=O\left(log_{2}a\cdot \sqrt{\frac{b}{a}}\right)$. Thus, the distance matrix space needed for one leaf node is:

$$O(log_{2}a\cdot \sqrt{\frac{b}{a}\cdot }b)$$

For all leaf nodes it would be:

$$O\left(log_{2}a\cdot \sqrt{\frac{b}{a}\cdot }b\cdot \frac{\left|V\right|}{b}\right)=O(log_{2}a\cdot \sqrt{\frac{b}{a}\cdot }\left|V|\right)$$

For the BVCP tree:

$$SP\left(EFCtree\right)=O(\log_{2}^{2}a\cdot \frac{1}{a}\cdot \log_{a}\frac{\left|V\right|}{b}|V|+\log_{2}a\cdot \sqrt{\frac{b}{a}}|V|)$$

whereas $log_{2}a$, $\frac{b}{a}$ and $log_{2}^{2}a\cdot \frac{1}{a}$ can be ignored since they are considered as small constants.

To conclude, the over space overhead of BVCP is

(1) $$\begin{array}{c} \begin{array}{c} SP(BVCP)\;\\ =SP(EFCgraph)+SP(EFCtree)\;\\ =O(|V|\log(|V|)\;+\;|E|) \end{array} \end{array}$$

BVCP accepts online requests and efficiently places the VNF chain in the intelligent transportation network. BVCP first checks whether the latency constraint can be met. If not, BVCP raises an error and informs the higher code logic. Concretely, BVCP transfers the latency check problem as the shortest path length finding problem.

For cases of the constraints can be satisfied, BVCP performs a linear search in the border set of the shortest path between two endpoints. The placement of VNFs on the Edge and Fog layer s would reduce the workload of the upper layers. It would also shorten the communication latency, which is of significant importance for transportation-related applications. Hence, BVCP gives low level-layers higher priority.

**Figure 5:**
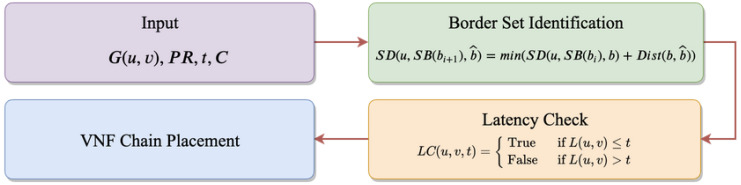
The flowchart of the BVCP algorithm.

Algorithm 1 shows the pseudo-code of the BVCP algorithm. We will show that the time complexity of the BVCP algorithm is O(|V|). The relationship of these algorithms and their input and output are shown in Figure 5.
**Algorithm 1:** BVCP algorithm 
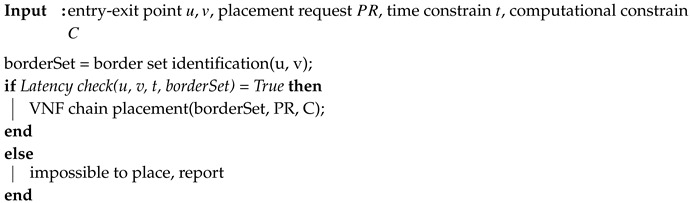


#### 3.2.5. Border Set Identification

To identify $b_{1},b_{2},\ldots ,b_{m}$, dynamic programming is used. Let SD(x,G,y) be the shortest path distance from a node *x*, to a hypervisor *y* of graph *G*. Since $leaf\left(u\right)=SB\left(b_{1}\right)$, $for\;b\;\in B(SB\left(b_{1}\right))$ we have:

(2) $$\begin{array}{c} SD\left(u,\;SB\left(b_{1}\right),\;b\right)\;=\;Dist\left(u,\;b\right) \end{array}$$

where Dist(u,b) can be directly obtained from the distance matrix in the leaf node.

Now we move forward, for $\hat{b}\in B\left(SB\left(b_{i+1}\right)\right)$:

(3) $$\begin{array}{c} \begin{array}{c} SD\left(u,SB\left(b_{i+1}\right),\hat{b}\right)=min\left(SD\left(u,SB\left(b_{i}\right),b\right)+Dist\left(b,\hat{b}\right)\right)\\ for\;b\in B(SB\left(b_{i}\right)) \end{array} \end{array}$$

where $Dist(b,\;\hat{b})$ can be directly obtained from the distance matrix in the non-leaf node and $SD(u,SB\left(b_{i}\right),b)$ is already computed in the last step.

**Figure 6:**
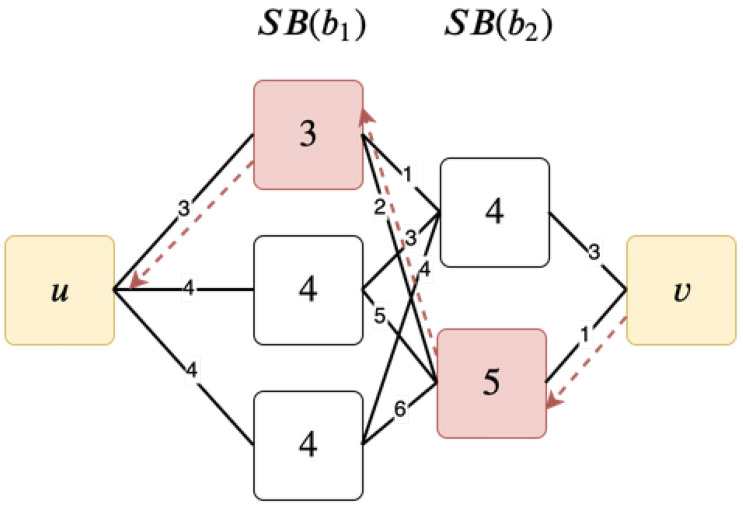
An example of border identification.

After SD is computed, we can easily go backward from *v* to trace back $b_{1},b_{2},\ldots ,b_{m}$. Figure 6 illustrates a simple border identification example. The black lines indicate the SD computation described above, and the red arrows show how to identify $b_{1},b_{2},\ldots ,b_{m}$ starting from *v*.

Algorithm 2 shows the pseudo-code of border set identification.
**Algorithm 2:** Border set identification 
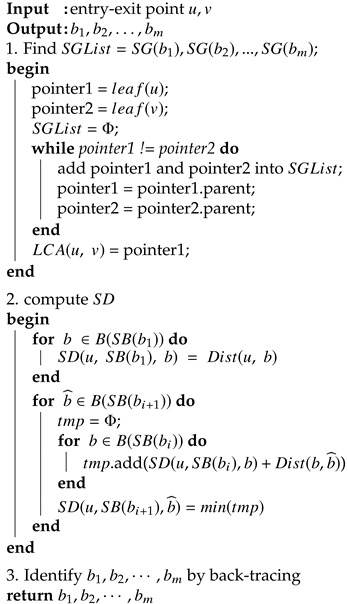


#### 3.2.6. Latency Check

The latency check problem can be modeled as the shortest path finding problem. Let function LC(u,v,t) be the latency check function which returns a Boolean value, L(u,v) be the shortest path function that returns the network latency, we have:

(4) $$\begin{array}{c} LC\left(u,\;v,\;t\right)\;=\;\left\{\begin{array}{c} True\;\;\;if\;L(u,\;v)\;\leq \;t\\ False\;\;if\;L(u,\;v)\;>\;t \end{array}\right. \end{array}$$

To implement L(u,v) efficiently, the algorithm utilizes the border hypervisors. It is clear that for (u,v) in different leaf nodes of a BVCP tree, the shortest path must at least contain two border nodes, one from the subgraph of *u* and another from the subgraph of *v*.

Formally, if $v\in G_{1},u\in G_{2}$, $\exists b_{1},b_{m}$ such that $b_{1}\in G_{1},\;b_{m}\in G_{2},b_{1}\in SP\left(u,\;v\right),\;b_{m}\in SP\left(u,\;v\right)$ where SP(u,v) is the shortest path between (u,v).

Hence, SP(u,v) can be decomposed into three parts:

- $u\to b_{1}$: From *u* to the border of $G_{1}$.
- $b_{1}\to b_{2}\to \cdots \to b_{m}$: From the border of $G_{1}$ to the border of $G_{2}$ through multiple intermediate subgraphs. $b_{1},b_{2},\ldots ,b_{m}$ is called the border set of the shortest path.
- $b_{m}\to v$: From the border of $G_{2}$ to *v*.

Formally, let leaf(u) be the leaf node that contains hypervisor *u*, the shortest path SP(u,v) between *u* and *v* must jump through a list of BVCP tree nodes, $SG\left(b_{1}\right)=leaf\left(u\right),SG\left(b_{2}\right),\ldots ,SG\left(b_{m}\right)=leaf\left(v\right)$.

**Figure 7:**
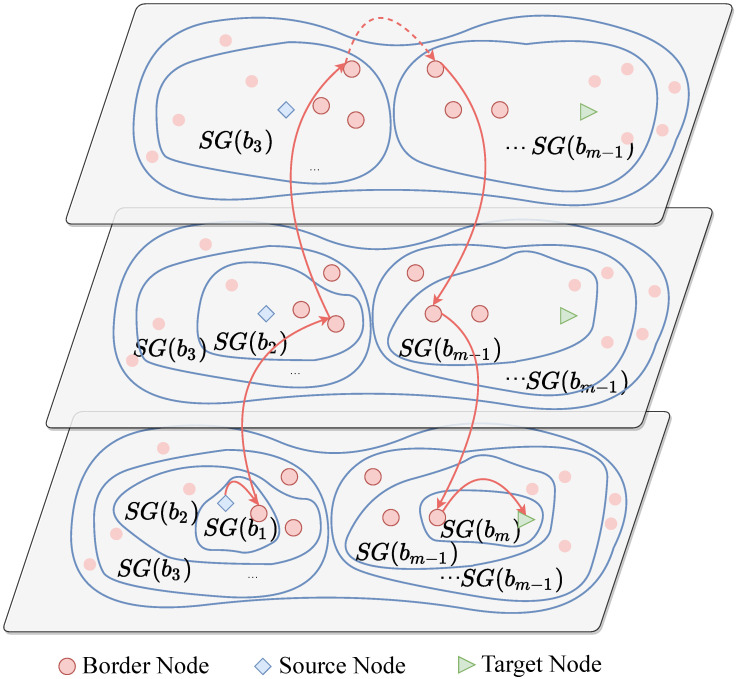
An illustration of the shortest path finding in the BVCP algorithm.

Figure 7 is an illustration of the shortest path finding in the BVCP algorithm.

### 3.3. Algorithm Procedure

Algorithm 3 shows the pseudo-code of latency check. Dist(x,y) is a matrix lookup function, it returns the shortest path distance between node *x* and *y*, given the condition that *x* and *y* is in the same subgraph(i.e. BVCP tree node) and can be found in the corresponding distance matrix.

$b_{1},b_{2},\ldots ,b_{m}$ are previously identified by the border set identification function BSI and passed to latency check as a parameter. The border set identification function BSI is discussed in the next subsection. BSI returns the border set of the shortest path between two nodes.
**Algorithm 3:** Latency check 
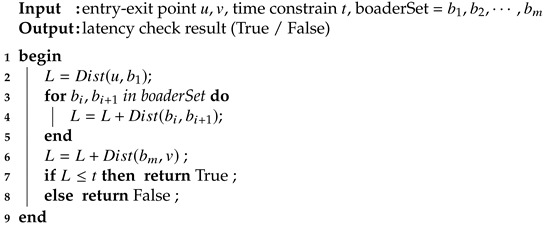


#### 3.3.1. VNF Chain Placement

Notice that the BVCP algorithms searches border hypervisors on the shortest path, $b_{1},b_{2},\ldots ,b_{m}$ is exactly the targets on which the computational constrain *C* is checked. Any border hypervisor that satisfies the constrain (denote as $S(b,\;C_{i})=True$) can be selected to place the VNF.

Algorithm 4 shows the pseudo-code of the VNF chain placement of BVCP.
**Algorithm 4:** VNF chain placement 
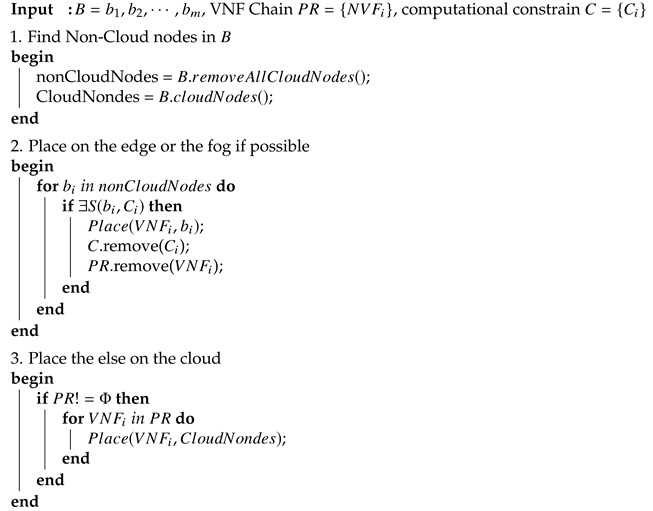


#### 3.3.2. Time Complexity

BVCP is an efficient VNF chain placement method and its time complexity is:

(5) $$\begin{array}{c} \begin{array}{c} T(BVCP)=\\ T(algorithm2)+T(algorithm3)+T(algorithm4) \end{array} \end{array}$$

where *T* is the time complexity. The time complexity of Algorithms 2–4 are discussed as follows: 

**Algorithm 2**

Since all the leaf nodes are at the same level, LCA(u,v) and $SG\left(b_{1}\right),SG\left(b_{2}\right),\ldots ,SG\left(b_{m}\right)$ can be found by alternately relocate two pointers initialized with *u* and *v* to its parent tree node until they point to the same node. This is linear to the height of the tree, hence is $O(\log_{a}\frac{\left|V\right|}{b}\;)$.

The average border number of a tree node at level *i* is $O(log_{2}a\cdot \sqrt{\frac{\left|V\right|}{a^{i+1}}})$. To locate a border hypervisor, the time complexity is square to it hence is $O(log_{2}^{2}a\cdot \frac{\left|V\right|}{a^{i+1}})$. The worst case is that the path goes through the root tree node and two tree nodes are checked each layer. Thus, the time complexity of Algorithm 2 is:

(6) $$\begin{array}{c} \begin{array}{c} T(algorithm2)\\ =\sum_{i}\;O\left(log_{2}^{2}a\cdot \frac{\left|V\right|}{a^{i+1}}\right)+O\left(\log_{a}\frac{\left|V\right|}{b}\;\right)\\ =O(|V|) \end{array} \end{array}$$

**Algorithm 3**

There is only one for-loop in Algorithm 3 and the time complexity of it is linear to the size of the border set which is |V| in the worst case.

Thus, the time complexity of Algorithm 3 is:

(7) $$\begin{array}{c} \begin{array}{c} T(algorithm3)\;=\;O(|V|) \end{array} \end{array}$$

**Algorithm 4**

The time complexity of scanning the border set is linear to the size of the border set which in the worst case is |V|.

Hence, the time complexity of Algorithm 4 is:

(8) $$\begin{array}{c} \begin{array}{c} T(algorithm4)\;=\;O(|V|) \end{array} \end{array}$$

Since latency check, boader set identification and VNF placement are executed only once in BVCP, the time complexity of BVCP is:

(9) $$\begin{array}{c} \begin{array}{c} T(BVCP)\\ =T(algorithm2)+T(algorithm3)+T(algorithm4)\\ =O(|V|)+O(|V|)+O(|V|)\\ =O(|V|) \end{array} \end{array}$$

## 4. Experiment Results

The proposed BVCP method is evaluated in a proof-of-concept emulated system. Each hypervisor is implemented as a docker container [30]. Docker is an open source, OS level virtualization tool designed to make application deployment easier. It allows us to implement virtual hypervisors conveniently.

A wide set of randomly generated networks are used to apply the algorithm. Interconnection between hypervisors are created by open-source complex network manipulation package NetworkX [31]. The generated networks are composed by a range of 100 to 5000 hypervisors, and each hypervisor has a degree ranged from 2 to 5. Placement requests of different VNF chain length range from 2 to 5 are generated randomly, endpoint edge hypervisors are also randomly select. Time constrain is set to 15 ms to 100 ms, CPU and memory constrains are set to 0.25 to 1 CPU core and 16 MB to 256 MB per VNF respectively.

The experiment is conducted on an Ubuntu 18.04 Linux machine equipped with 2 x Intel Xeon E5-2620v4 (16 cores), 128 GB RAM.

The performance evaluation in the experiment consists of two parts:

- the average placement time
- the average topology update time once an edge hypervisor changed from one subgraph to another subgraph

**Figure 8:**
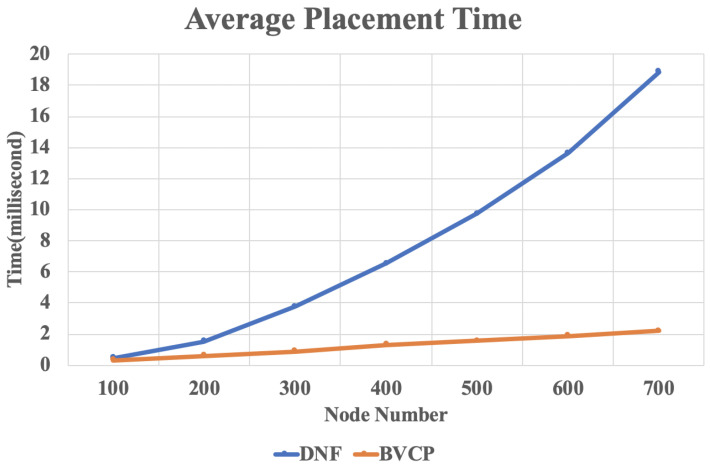
The result of average placement time.

Figure 8 shows the average placement time of the proposed BVCP algorithm and the baseline algorithm DNF [10] for different number of nodes in the network. It can be observed from the illustration that the performance of the proposed algorithm has a significant advantage comparing to the baseline algorithms for large networks. This result agrees with the theoretical analysis above that the proposed BVCP algorithm is scalable for large networks. With the growth of nodes in the network, the average placement time of the proposed BVCP algorithm grows linearly. Such property will make great difference in real-world applications.

**Figure 9:**
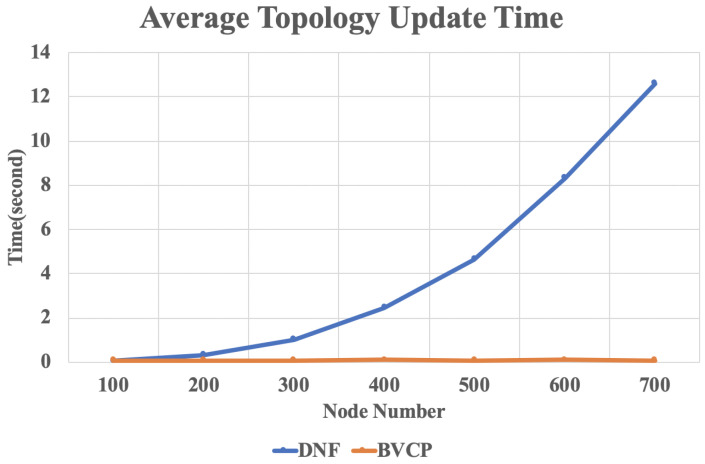
The result of average topology update time.

Figure 9 shows the average topology update time of the proposed BVCP algorithm and the baseline algorithm for different number of nodes in the network. It can be seen that with the scale of the network grows, the update time of the proposed BVCP algorithm remains a small constant. However, for the baseline algorithm, it grows exponentially. Considering that subgraph shifting is common in the smart transportation networks, the results justify the design of BVCP graph and BVCP tree.

After the performance evaluation part, a comparative experiment was conducted to estimate the impact of different factors on VNF chain placement. In order to focus on the practical significance of VNF chain replacement, the effects of two factors, subgraph size and network connectivity, on VNF chain replacement are evaluated.

**Figure 10:**
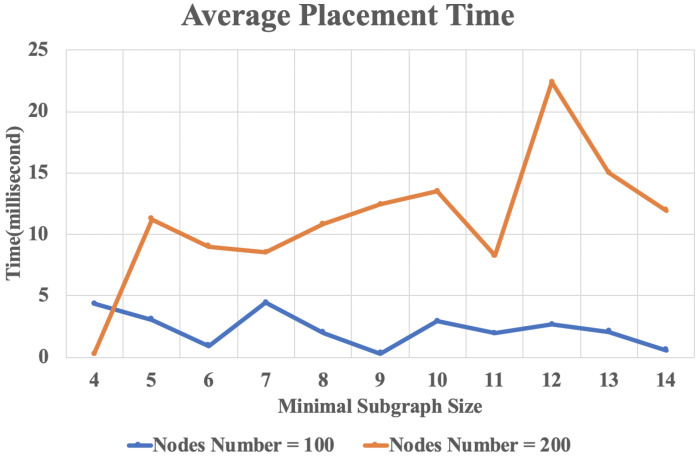
The result of average placement time with different subgraph size.

In practical applications, by obtaining the optimal partition size of the subgraph, network infrastructure administrator can get the optimal division of the cloud-fog layer structure of the VNF, thereby optimizing the structure of the network. Hence the VNF chain replacement may consumes less time. Figure 10 shows the average placement time when using different subgraph size. It can be seen that although the size of the minimal subgraph has an effect on the time of VNF chain replacement, it is not a decisive factor. For different number of nodes, the minimal subgraph size that minimizes the replacement time is not constant.

**Figure 11:**
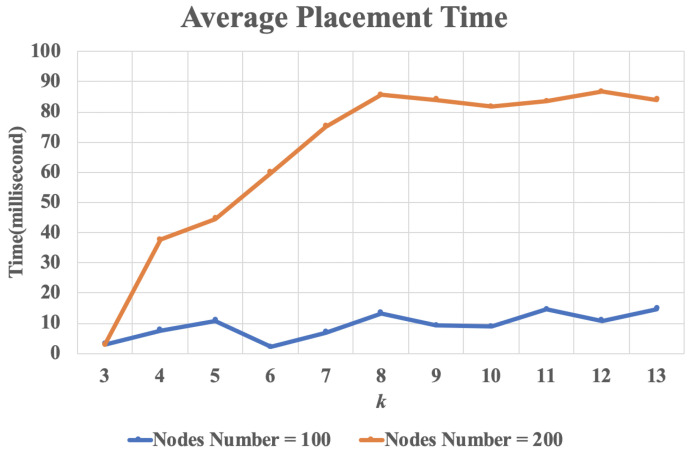
The result of the average placement time with different *k* value.

To test the impact of network connectivity on the VNF chain replacement for BVCP method, we used a *k*-regular graph as the randomly generated network graph. Keeping the number of nodes constant, by changing the value of *k*, the performance data of the algorithm under different connectivity can be obtained. When the value of *k* is larger, the more edges of the nodes are connected, the stronger the connectivity of the network. Figure 11 shows the average placement time with different *k* value when minimal subgraph size is 4. It can be seen that BVCP has better performance when the value of *k* is small. Considering practical applications, most VNF networks are small-world network, and each node is only connected to a few nodes in the network. Therefore, in practical applications, the performance of BVCP will be more prominent.

## 5. Conclusions

The ramifications of the Internet of Things have reshaped the intelligent transportation by revolutionizing the way edge devices interact with transportation services. Fog computing has been proven to be an effective solution for large scale transportation networks. Thus, an Edge–Fog–Cloud interplay paradigm is proposed for the evolving topology of IoT networks in intelligent transportation and it is mapped to a graph. The graph computing has demonstrated its capabilities in managing large-scale network topologies. Inspired by the good property of graph theory, a novel VNF chain placement algorithm called BVCP is designed, utilizing the salient features of subgraphs and border hypervisors to enable flexible deployments of service chains on dynamically evolving IoT networks. According to the experimental results, BVCP outperforms the state-of-the-art VNF chain placement algorithm. It is believed that BVCP fits exactly in large scale IoT of intelligent transportations to empower quick intelligent transportation applications and services.
